# Ultrasound-Assisted, Catheter-Directed Thrombolysis for Acute Intermediate/High-Risk Pulmonary Embolism: Design of the Multicenter USAT IH-PE Registry and Preliminary Results

**DOI:** 10.3390/jcm13020619

**Published:** 2024-01-22

**Authors:** Claudia Colombo, Nicolò Capsoni, Filippo Russo, Mario Iannaccone, Marianna Adamo, Giovanna Viola, Ilaria Emanuela Bossi, Luca Villanova, Chiara Tognola, Camilla Curci, Francesco Morelli, Rossella Guerrieri, Lucia Occhi, Giuliano Chizzola, Antonio Rampoldi, Francesco Musca, Giuseppe De Nittis, Mario Galli, Giacomo Boccuzzi, Daniele Savio, Davide Bernasconi, Luciana D’Angelo, Andrea Garascia, Alaide Chieffo, Matteo Montorfano, Fabrizio Oliva, Alice Sacco

**Affiliations:** 1 1st Division of Cardiology, De Gasperis Cardio Center, ASST Grande Ospedale Metropolitano Niguarda, 20162 Milan, Italy; claudia.colombo@ospedaleniguarda.it (C.C.); giovanna.viola@ospedaleniguarda.it (G.V.); luca.villanova@ospedaleniguarda.it (L.V.); fabrizio.oliva@ospedaleniguarda.it (F.O.); 2 Department of Emergency Medicine, ASST Grande Ospedale Metropolitano Niguarda, 20162 Milan, Italy; nicolo.capsoni@ospedaleniguarda.it (N.C.); ilariaemanuela.bossi@ospedaleniguarda.it (I.E.B.); camilla.curci@ospedaleniguarda.it (C.C.); rossella.guerrieri@ospedaleniguarda.it (R.G.); 3 Interventional Cardiology Unit, IRCCS San Raffaele Hospital, 20132 Milan, Italy; russo.filippo@hsr.it (F.R.); chieffo.alaide@hsr.it (A.C.); montorfano.matteo@hsr.it (M.M.); 4 Division of Cardiology, San Giovanni Bosco Hospital, 10154 Turin, Italy; mario.iannaccone@hotmail.it (M.I.); giacomoboccuzzi@aslcittaditorino.it (G.B.); 5 Institute of Cardiology, ASST Spedali Civili di Brescia, Department of Medical and Surgical Specialties, Radiological Sciences, and Public Health, University of Brescia, 25123 Brescia, Italy; mariannaadamo@hotmail.com; 6 4th Division of Cardiology, De Gasperis Cardio Center, ASST Grande Ospedale Metropolitano Niguarda, 20162 Milan, Italy; chiara.tognola@ospedaleniguarda.it (C.T.); lucia.occhi@ospedaleniguarda.it (L.O.); francesco.musca@ospedaleniguarda.it (F.M.); 7 Department of Interventional Radiology, Niguarda Cà Granda Hospital, 20142 Milan, Italy; francesco.morelli@ospedaleniguarda.it (F.M.); antoniogaetano.rampoldi@ospedaleniguarda.it (A.R.); 8 Cardiac Catheterization Laboratory and Cardiology, ASST Spedali Civili di Brescia, 25121 Brescia, Italy; gchizzola@hotmail.com; 9 Cardiovascular Interventional Unit, Cardiology Department, S. Anna Hospital, 10126 Como, Italy; giuseppe.denittis@asst-lariana.it (G.D.N.); mario.galli@asst-lariana.it (M.G.); 10 Department of Interventional Radiology, San Giovanni Bosco Hospital, 10154 Turin, Italy; daniele.savio@aslcittaditorino.it; 11 Bicocca Bioinformatics Biostatistics and Bioimaging (B4) Center, School of Medicine and Surgery, University of Milano-Bicocca, 20126 Bicocca, Italy; davide.bernasconi@unimib.it; 12 Department of Clinical Research and Innovation, ASST Grande Ospedale Metropolitano Niguarda, 20162 Milan, Italy; 13 2nd Division of Cardiology, De Gasperis Cardio Center, ASST Grande Ospedale Metropolitano Niguarda, 20162 Milan, Italy; luciana.dangelo@ospedaleniguarda.it (L.D.); andrea.garascia@ospedaleniguarda.it (A.G.)

**Keywords:** pulmonary embolism, ultrasound assisted, catheter-directed thrombolysis, pulmonary hypertension

## Abstract

Catheter-based revascularization procedures were developed as an alternative to systemic thrombolysis for patients with intermediate-high- and high-risk pulmonary embolisms. USAT IH-PE is a retrospective and prospective multicenter registry of such patients treated with ultrasound-facilitated, catheter-directed thrombolysis, whose preliminary results are presented in this study. The primary endpoint was the incidence of pulmonary hypertension (PH) at follow-up. Secondary endpoints were short- and mid-term changes in the echocardiographic parameters of right ventricle (RV) function, in-hospital and all-cause mortality, and procedure-related bleeding events. Between March 2018 and July 2023, 102 patients were included. The majority were at intermediate–high-risk PE (86%), were mostly female (57%), and had a mean age of 63.7 ± 14.5 years, and 28.4% had active cancer. Echocardiographic follow-up was available for 70 patients, and in only one, the diagnosis of PH was confirmed by right heart catheterization, resulting in an incidence of 1.43% (CI 95%, 0.036–7.7). RV echocardiographic parameters improved both at 24 h and at follow-up. In-hospital mortality was 3.9% (CI 95%, 1.08–9.74), while all-cause mortality was 11% (CI 95%, 5.4–19.2). Only 12% had bleeding complications, of whom 4.9% were BARC ≥ 3. Preliminary results from the USAT IH-PE registry showed a low incidence of PH, improvement in RV function, and a safe profile.

## 1. Introduction

Acute pulmonary embolism (PE) is a potentially life-threatening disease with annual incidence rates ranging from 39 to 115 per 100,000 population [1]. It spans a wide spectrum of clinical outcomes, and it is classified according to the 30-day risk of mortality based on clinical, imaging, and laboratory findings [2].

Reperfusion therapy with systemic thrombolysis is the treatment of choice in high-risk PE; however, only a minority of such patients receive this treatment due to comorbidities or the risk of major hemorrhagic side effects [3]. Intermediate-risk PE accounts for 45% to 65% of PE, with a short-term mortality of around 3% [4]. Systemic thrombolysis is not generally recommended for this group as hemorrhagic complications can outweigh the benefits; however, patients at higher risk within this group (i.e., intermediate–high-risk patients) present a short-term mortality of around 12%, suggesting the need for more aggressive treatment [4]. 

Surgical embolectomy or catheter-directed therapy (CDT) are alternatives to systemic thrombolysis in such patients. However, the former is not usually immediately available, thus rendering the latter the treatment of choice in emergent contexts. 

Minimally invasive reperfusion treatments, such as CDT strategies, are currently suggested by ESC guidelines (class IIa, LOE C) for patients with high-risk PE and contraindications for systemic thrombolysis, or patients with intermediate–high-risk PE and hemodynamic deterioration while on anticoagulation [2]. Due to a growing interest in this field, several different techniques have emerged and are currently available. One of these treatments is ultrasound-assisted thrombolysis (USAT), which allows local infusion of a low-dose thrombolytic agent facilitated by ultrasounds. Using this procedure, ultrasound causes reversible disaggregation and separation of uncross-linked fibrin fibers, increasing thrombus permeability for thrombolytic drugs [5,6,7]. 

Data regarding USAT are promising in terms of safety and efficacy, showing acute improvement in RV function and a reduction in pulmonary artery systolic pressure (PASP), without major hemorrhagic complications [8,9]. There is, however, insufficient data on mid- and long-term outcomes of patients receiving such therapy and a lack of evidence to support the widespread adoption of CDT for acute PE. 

This gap in the evidence was the reason why we designed the USAT IH-PE study, which aims to investigate specific clinical outcomes related to the procedure.

In the present paper, we sought to present retrospective analyses as well as the rationale and design of the prospective USAT IH-PE study. 

## 2. Materials and Methods

### 2.1. Trial Design

The USAT IH-PE was a single-center retrospective study recently upgraded into a multicenter prospective registry including all consecutive patients hospitalized in 5 centers (ASST Grande Ospedale Metropolitano Niguarda in Milan, IRCCS Ospedale San Raffaele in Milan, Ospedale San Giovanni Bosco in Turin, Spedali Civili in Brescia, and Ospedale Sant’Anna in Como) with a diagnosis of intermediate-high- and high-risk PE treated with the Ekosonic^®^ device (Boston Scientific Corporation, Natick, MA, USA). Both retrospective and prospective studies were coordinated by the ASST Grande Ospedale Metropolitano Niguarda, in agreement with the Helsinki II declaration, and approved by the Ethical Committee of the ASST Grande Ospedale Metropolitano Niguarda (proc S00079/2022, approval date 12 December 2022) and by the competent committees for all the participating centers.

Our manuscript includes the analysis of retrospective data from 102 patients admitted for acute intermediate-high- and high-risk PE between March 2018 and July 2023 and treated with EKOS^TM^ at ASST Grande Ospedale Metropolitano Niguarda prior to the expansion of the retrospective registry. Trial recruitment will be completed across the 5 Italian hospital sites in November 2026. Participants will be followed until the end of June 2027. We plan to report the results by December 2027. 

The primary endpoint was the incidence of pulmonary hypertension (PH) at follow-up. PH was defined by mean pulmonary artery pressure ≥ 20 mm Hg at rest during right heart catheterization [10] in patients who presented PASP ≥ 40 mmHg at the echocardiographic follow-up. Secondary efficacy endpoints were short- and mid-term changes in echocardiographic parameters of RV function (tricuspid annular plane systolic excursion (TAPSE), the RV/left ventricle (LV) ratio, pulmonary artery systolic pressure (PASP), TAPSE/PASP ratio), and in-hospital mortality and all-cause mortality. The secondary safety endpoint was procedure-related bleeding according to the Bleeding Academic Research Consortium (BARC) criteria, where major bleeding was defined as ≥IIIa [11]. PE risk was defined according to the current classification provided by ESC Guidelines, dividing patients into low, intermediate-low, intermediate-high, and high risk [2]. For the purpose of the current analysis, we included both intermediate-high- and high-risk patients. Other inclusion criteria were: symptom onset within the previous 14 days associated or not with deep venous thrombosis (DVT), confirmation of PE by contrast-enhanced computed tomography with thrombotic occlusion in at least one of the main pulmonary arteries or one of the proximal lower lobe arteries, RV dysfunction at echocardiography (defined as at least one of the following: RV/LV ratio > 1, TAPSE ≤ 16 mm, TAPSE/PASP < 0.4), and intermediate to high probability of PH (PASP > 40 mm Hg). Exclusion criteria were age < 18 years old, patients unable to give informed consent, pregnancy, fibrinolytic drugs in the previous 4 days, bleeding diathesis and/or known bleeding disorder, low platelet count (<100.000 µL), gastrointestinal bleeding in the previous 3 months, active cancer with expected survival < 6 months, and advanced chronic kidney disease (CKD, defined as estimated glomerular filtration rate < 30 mL/min/1.73 m^2^ or patient on dialysis). 

The EKOS^TM^ system is implanted with fluoroscopic guidance. For unilateral PE in one main or proximal lobar pulmonary artery, one catheter was placed in the involved vessel. With bilateral PE in the main or proximal lobar pulmonary arteries, two catheters were placed, one in each of the involved vessels. The site of venous access (jugular or femoral) was at the discretion of the treating physician. Over 10 h, 10 mg of alteplase per catheter was continuously administered at a rate of 1 mg/h. At the same time, an intravenous infusion of unfractioned heparin (UFH) was initiated with target aPTT 50–70 s. After 10 h, the therapy was stopped and the EKOS^TM^ catheter was removed.

In-hospital and mid-term (3–6 months) outcomes were evaluated from hospital records and follow-up assessments. Follow-up consisted of an ambulatory visit with clinical assessment, 12-lead ECG, and transthoracic echocardiography. The timing of follow-up after discharge was planned considering the echocardiographic probability of pulmonary hypertension (PH), estimated with an echocardiogram performed during hospitalization. Such probability may be judged as high, intermediate, or low according to ESC PH guidelines [10]. If the probability was judged as low, follow-up was performed at 6 months, while in the case it was judged as intermediate or high, follow-up occurred at 3 months with echocardiography and PH ambulatory consultation. In the case of the persistence of an intermediate or high probability of PH at follow-up, a perfusion imaging exam (V/Q scan) and right heart catheterization (RHC) were performed to confirm the diagnosis of chronic thrombo-embolic pulmonary hypertension (CTEPH). 

### 2.2. Statistical Analysis

The distribution of continuous variables was described as mean  ±  standard deviation or median (interquartile range (IQR))

The incidence of PH, in-hospital death, and mortality during follow-up were computed with 95% confidence intervals. 

The distribution of TAPSE, PASP, the RV/LV ratio, and the TAPSE/PASP ratio was compared between three time points (T0, T24, and T-FU) using the Wilcoxon signed-rank test. The percentage of patients with TAPSE ≤ 16 mm and/or PASP ≥ 40 mmHg and/or RV/LV > 1 was also calculated for the three time points and compared pairwise using the McNemar test.

Statistical analyses were performed using SPSS software (version 25.0, SPSS Inc., Chicago, IL, USA) and R statistical software version 4.2.2 (R Foundation for Statistical Computing, Vienna, Austria).

## 3. Results

Between March 2018 and July 2023, 102 patients affected by PE at intermediate-high risk and high risk were included. The majority of patients (86%, n = 88) had intermediate-high-risk PE. An overview of the patients’ baseline characteristics is provided in Table 1. Briefly, the mean age was 63.7 ± 14.5 years, with a female prevalence (56.9%, n = 58) and a high incidence of active cancer (28.4%, n = 29). Systemic thrombolysis was contraindicated for 19% of patients, of which 14% presented an absolute contraindication. 

The majority of patients (n = 77.8%) at CT scan presented a bilateral involvement of the main pulmonary arteries, 7 patients (7%) had a unilateral involvement of one of the main pulmonary arteries, and 15 patients (15%) had a bilateral lobar involvement of the pulmonary arteries. 

The echocardiographic evaluation at the time of PE diagnosis showed signs of RV impairment, such as a mean TAPSE of 16 ± 3 mm, a mean RV/LV ratio of 1 ± 0.2, a mean TAPSE/PASP ratio of 0.3 ± 0.1, and a mean PASP of 48 ± 13 mmHg. 

### 3.1. Outcomes

#### 3.1.1. The Primary Endpoint

For 32 patients, no echocardiographic follow-up data could be obtained after discharge, and they were excluded from the analysis. This resulted in a total of 70 patients who were analyzed for the primary outcome. Only two patients had a PASP ≥ 40 mmHg at the echocardiographic evaluation and, of those, in only one patient, the diagnosis of PH was confirmed by right heart catheterization. Hence, the risk of incidence of PH was 1.43% (CI 95%, 0.036–7.7), and the rate of incidence was 3.31 (CI 95%, 0.08–17.42) × 100 year-person. 

#### 3.1.2. Secondary Efficacy Endpoint

The changes in echocardiographic parameters of RV function are summarized in Table 2. At 24 h, the mean TAPSE was 19.5 ± 3.6 mm, the mean PASP was 38 ± 11 mmHg, the mean RV/LV ratio was 0.9 ± 0.2, and the mean TAPSE/PASP was 0.5 ± 0.2. The median time of echocardiographic follow-up was 144 days from discharge [IQR = 90, 207]. At follow-up, the mean TAPSE was 22.5 ± 3.5 mm, the mean PASP was 31 ± 7 mmHg, the mean RV/LV ratio was 0.8 ± 0.1, and the mean TAPSE/PASP ratio was 0.8 ± 0.2.

**Figure 1:**
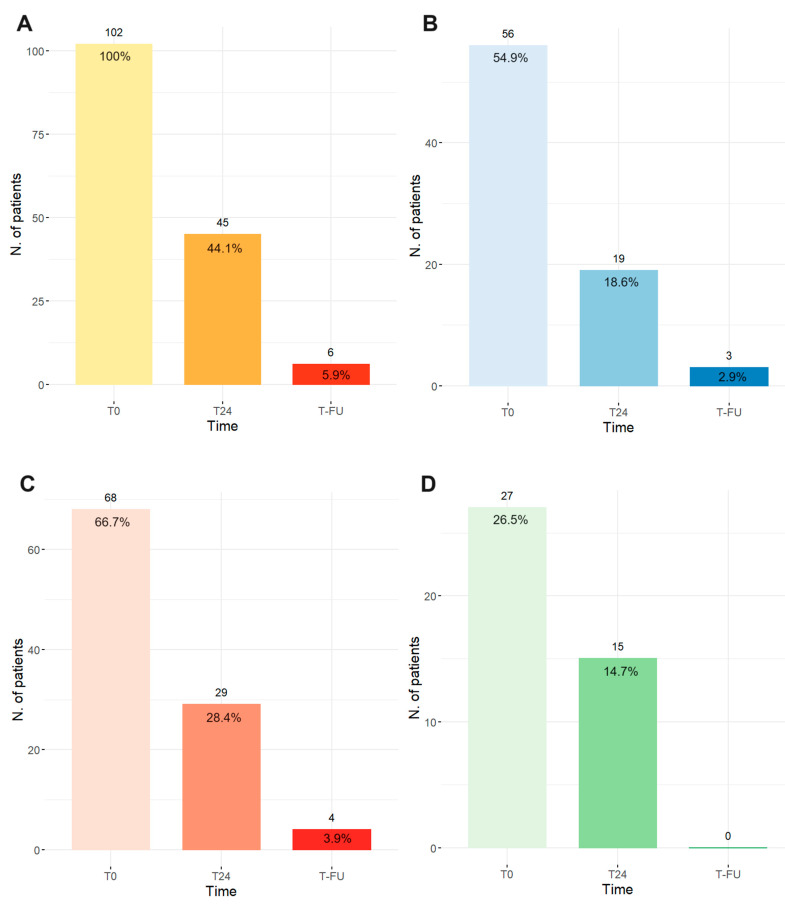
Echocardiographic parameters of RV function at T0 (hospital admittance), T24 (after 24 h post-EKOS), and T-FU (3–6 months). (**A**) TAPSE ≤ 16 mm and/or PASP ≥ 40 mmHg and/or RV/LV > 1, (**B**) TAPSE ≤ 16 mm, (**C**) PASP ≥ 40 mmHg, and (**D**) RV/LV > 1. All pairwise comparisons between time points have *p* < 0.001 except RV/LV > 1 T0 vs. T24 with *p* = 0.038 according to the McNemar test.

At the first echocardiographic assessment 24 h after the treatment (T24), 44.1% of patients showed signs of RV failure (TAPSE ≤ 16 mm and/or PASP ≥ 40 mmHg and/or RV/LV > 1), while at follow-up, only 5.9% showed these signs (Figure 1A). Figure 1B–D shows singularly the number of patients with TAPSE ≤ 16 mm, PASP ≥ 40 mmHg, and RV/LV > 1. 

**Figure 2:**
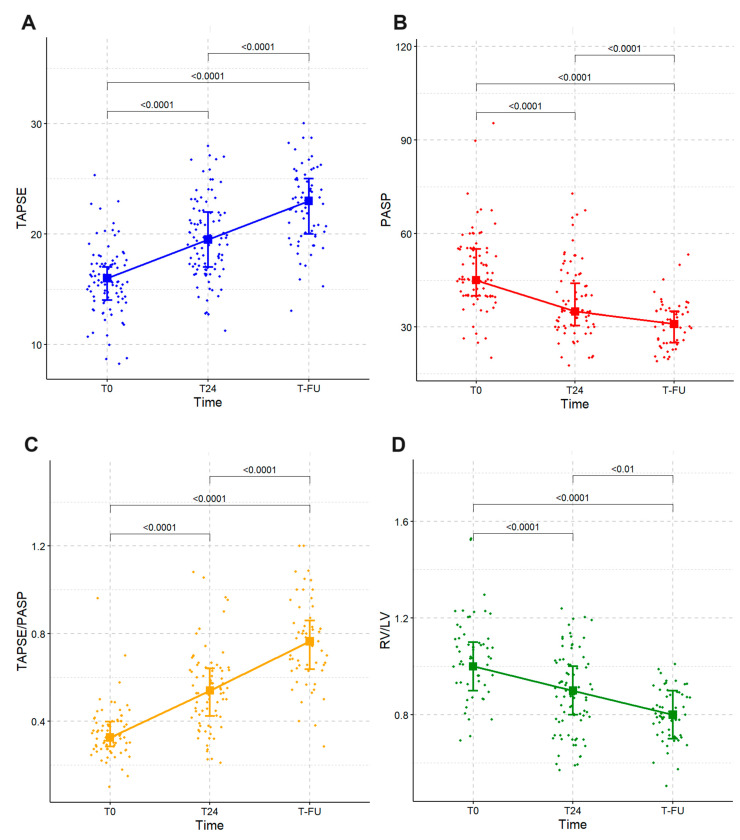
Distribution of TAPSE (**A**), PASP (**B**), TAPSE/PASP (**C**), and RV/LV (**D**) at the 3 considered time points.

The distribution of TAPSE (A), PASP (B), TAPSE/PASP (C), and RV/LV (D) at the three considered time points in terms of median and interquartile range (Q1–Q3) is depicted in Figure 2. The Wilcoxon signed-rank test was applied to compare the distribution of values between time points (i.e., T0 vs. T24, T0 vs. T-FU, T24 vs. T-FU) showing a significant improvement in all four parameters (Figure 2).

#### 3.1.3. Mortality

Four patients died during the index hospitalization, resulting in an in-hospital mortality of 3.9% (CI 95%, 1.08–9.74). Ten patients died during follow-up (11%): none from complications related to PE but mainly due to neoplastic disorders (n = 7) or unknown causes (n = 3). The incidence of all-cause mortality during follow-up was 11% (CI 95%, 5.4–19.2).

#### 3.1.4. Secondary Safety Endpoint

The majority of patients (88%) had no bleeding complications. Of the patients presenting any bleeding, only 4.9% were classified as major bleeding (BARC ≥ IIIa), with no reported intracranial or fatal bleeding. 

## 4. Discussion

The main findings of our study can be summarized as follows: (i) the incidence of PH following USAT is low, (ii) echocardiographic parameters of RV function improve both at short- (24 h) and mid-term (3–6 months) follow-up, and (iii) USAT is a safe procedure with a low rate of bleeding events. 

The baseline characteristics of our population are comparable with the existing literature regarding this subject in terms of age, female sex prevalence, and BMI [8,9,12,13]. 

The main studies in the literature concerning CDT focused primarily on the short-term outcomes of efficacy (i.e., improvement in RV function, defined with echocardiography) and safety. This is reasonable, considering the immediate and evident benefits of a local therapy that targets the source of subsequent RV impairment.

Indeed, a recent systematic review [4], which was aimed at including randomized controlled trials (RCTs) of CDT for the treatment of high-risk and intermediate-risk acute PE, identified only one RCT in this specific setting [12]. Moreover, as pointed out by the authors, the majority of trials investigated surrogate echocardiographic outcomes without considering relevant clinical outcomes. Therefore, the purpose of our study was to attempt to fill this gap by investigating a relevant primary outcome, such as the development of PH, and extending all analyses to mid-term follow-up (3–6 months). Several RCTs on this topic are ongoing, among which the STRATIFY and the HI-PEITHO trials will specifically provide information regarding the incidence of PH at follow-up. However, only the HI-PEITHO trial requires confirmation of PH at RHC after finding elevated PASP at echocardiography, as recommended by the ESC guidelines [10]. In our study, the incidence of PH, defined as well by the confirmation at RHC of elevated PASP at echocardiography, was low (1.43%), with only one of the two patients with PASP ≥ 40 mmHg having the diagnosis confirmed at RHC. The correct diagnosis of PH at follow-up represents a fundamental aspect of PE patients, in order to identify those patients at risk of developing chronic thromboembolic pulmonary hypertension (CTEPH). Such a condition, with a reported cumulative incidence of between 0.1 and 9.1% in the first 2 years after a symptomatic PE event [14], is caused by the persistent obstruction of pulmonary arteries by organized thrombi and leads to secondary remodeling of the pulmonary microvascular bed and, consequently, RV function impairment. 

When we considered echocardiographic parameters of RV function, all improved after the USAT treatment, both at 24 h and at 3–6 months follow-up. 

Among all considered parameters, RV to pulmonary arterial coupling, expressed as the TAPSE/PASP ratio, deserves special consideration, given its validated prognostic role in several settings such as heart failure (HF) and PH [15,16,17,18]. By measuring the adaptation of the RV to its afterload, this parameter may help to detect pending RV failure. However, to the best of our knowledge, the TAPSE/PASP ratio in patients undergoing CDT has not been evaluated to date. In a retrospective analysis of 627 patients, Lyhne et al. found that a TAPSE/PASP ratio < 0.4 was an independent predictor of mortality in patients with PE [19]. In our study, we found a mean TAPSE/PASP ratio during the acute phase of 0.3 ± 0.1, which improved, 24 h after the end of USAT, up to a mean of 0.5 ± 0.2 and, at follow-up, up to a mean of 0.8 ± 0.2, which is a value close to the validated normal range in the literature (0.8–1.8) [20,21,22]. 

In a recent 2022 meta-analysis [23] of 65,589 patients with intermediate- and high-risk PE treated with CDT, in-hospital mortality was 6.4%, which is a reduction by half if compared with a previous meta-analysis published in 2009 (in-hospital mortality 13.6%) [24]. The authors explained such a reduction by the improvement in CDT techniques, the increased experience, and the establishment of pulmonary embolism response teams (PERTs) for risk stratification and appropriate patient selection for CDT. In our study, in-hospital mortality was 3.9% (CI 95%, 1.1–9.7), and this even lower event rate could be due to the smaller sample size and the specific focus on USAT. During follow-up, 10 patients (11%) died: none died due to complications related to PE but mainly due to neoplastic disorders or unknown causes. The risk of all-cause mortality was 11% (CI 95%, 5.4–19.2). 

Regarding the safety endpoint, the majority of patients (88%) had no bleeding complications. Of the patients presenting bleeding complications, only 4.9% were classified as major bleeding (BARC ≥ IIIa), with no reported intracranial or fatal bleeding. In the SEATTLE II study [8], the rate of major bleeding events was twice as high (10%), probably due to the different classification of bleeding events (GUSTO classification instead of BARC) and a higher prevalence of high-risk PE (21% vs. 14%). 

One aspect of particular interest in our study is the relatively high prevalence of active cancer, which was present in about one-third of our population. As is known from the literature [25], in cancer patients, PE is the second cause of death after death due to the cancer itself. In this specific subset of PE patients, the best therapeutic strategy is not standardized yet because of the coexisting increased risk of bleeding and recurrent thrombosis. Moreover, Weeda et al. [26] and Shalaby et al. [27] found that cancer patients are less likely to receive thrombolysis as compared with non-cancer patients (OR = 0.55, 95% CI (0.48–0.64), and OR = 0.68, 95% CI (0.64–0.72)), particularly in the case of metastatic disease, probably due to a general overestimation of the bleeding risk. Unfortunately, no data on the incidence of bleeding or mortality were reported in these two studies. The underuse of systemic thrombolysis has consequently led to a less accurate estimation of the risk of bleeding in cancer patients who undergo thrombolysis.

A small number of cancer patients were included in the studies of CDT, and the isolated outcomes of this specific subgroup were not assessed [8,9,12]. Therefore, in cancer patients, indications of CDT, as well as data on efficacy and safety, are lacking. Considering that almost one-third of our population is represented by cancer patients, the positive results, in terms of a low incidence of PH, favorable efficacy, and favorable safety, constitute important data if applied to a poorly represented population in other studies of CDT. 

Lastly, we also emphasize the relevant role of building solid “pulmonary Embolism Response Teams (PERTs)”, composed of multidisciplinary experts for the management of “severe” (high-risk and intermediate-high-risk) PE [28]. Institutions have developed PERTs to assist with choosing the best treatment strategy and evaluating the possible need for advanced therapies such as CDT. In every center involved in our study, the multidisciplinary team, mainly consisting of a cardiac intensivist, emergency department physician, and interventional radiologist, has proven to represent the best method to balance risks and benefits for each treatment option according to PE risk. 

The main limitations of our study include its mainly retrospective nature, as well as the absence of a control arm. Another limitation was the relatively small sample size, which resulted in performing the RHC eventually in only one patient. Moreover, all echocardiographic parameters were collected independently by each center without involving external validation by a core lab. 

## 5. Conclusions

The ultrasound-assisted CDT with EKOS system in patients with intermediate-high- and high-risk PE is associated with a low incidence of PH at follow-up, improvement in echocardiographic parameters of RV function, and a safe profile. The relatively high percentage of cancer patients included in our study, generally underrepresented in the studies conducted in this field, provides important information regarding efficacy and safety in this specific subset of patients. This study adds valuable information about relevant clinical and mid-term outcomes. Data from the ongoing prospective registry as well as the upcoming results from RCTs will better define the role of CDT in patients at intermediate-high- and high-risk PE and the effects on clinically relevant outcomes.

## Figures and Tables

**Table 1:** Baseline demographics, clinical characteristics, and laboratory (N = 102).

Baseline Demographics	
Female	58 (56.9)
Age	63.7 ± 14.5
Smoking current past	8 (7.8) 15 (14.7)
BMI	28.1 ± 5.2
Hypertension	58 (56.9)
Cancer	29 (28.4)
Previous DVP	21 (20.6)
Heart failure	6 (5.9)
CAD	6 (5.9)
Previous stroke	8 (7.8)
Previous PE	9 (8.8)
Previous DVP	21 (20.6)
Diabetes mellitus	13 (12.7)
Chronic kidney disease	11 (10.8)
Presenting symptoms	
Dyspnea	75 (73.5)
Chest pain	23 (22.5)
Syncope	20 (19.6)
Hemoptysis	0
Peripheral edema	8 (7.8)
Heart palpitations	4 (3.9)
Clinical presentation	
Systolic blood pressure, mm Hg	127 ± 24
Diastolic blood pressure, mmHg	76 ± 15
Heart rate, BPM	103 ± 19
Oxygen saturation, %	95 ± 4
First available PaO_2_/FiO_2_	244 ± 102
Sepsis	2 (2)
Neurological alteration	8 (8.1)
Laboratory	
Hemoglobin (g/dL)	12.9 ± 2
Platelets (n × 10^3^/mm^3^)	221 ± 79
WBC, ×10^3^/μL	12 ± 8
D-dimer, ng/mL	16.8 ± 11.1
Creatinine, mg/dL	1.1 ± 0.6
Troponin on admission ng/L	102.6 ± 111.4
NT-proBNP, ng/L	3948.7 ± 6374.8
Arterial lactate, mmol/L	2.5 ± 2
Characteristics of PE at CT scan	
Bilateral main pulmonary arteries	77 (75.5)
Unilateral main pulmonary arteries	7 (6.9)
Bilateral, lobar	15 (14.7)
Unilateral, lobar	3 (3.6)

Values are mean ± standard deviation or n (%). BMI, body mass index; BNP, brain natriuretic peptide; CAD: coronary artery disease; DVT: deep vein thrombosis; PE: pulmonary embolism; CT, computed tomography; NT-proBNP, N-terminal pro-BNP; Pao2, oxygen partial pressure at arterial gas analysis; WBC, white blood cell. BPM: beats per minute.

**Table 2:** The changes in echocardiographic parameters of RV function.

	T 0	T24	T-FU
TAPSE ≤ 16 mm TAPSE (mm) ΔTAPSE	56 (63) 16 ± 3	19 (19.8) 19.5 ± 3.6 3.4 ± 2.8	3 (4.5) 22.5 ± 3.5 6.2 ± 3.1
PASP ≥ 40 mmHg PASP (mmHg) ΔPASP	68 (83) 48 ± 13	29 (34) 38 ± 11 −9.9 ± 9.7	4 (7) 31 ± 7 −17.4 ± 9.6
RV/LV > 1 RV/LV ΔRV/LV	27 (48) 1 ± 0.2	15 (18) 0.9 ± 0.2 −0.2 ± 0.2	0 (0) 0.8 ± 0.1 −0.2 ± 0.1
TAPSE/PASP ΔTAPSE/PASP	0.3 ± 0.1	0.5 ± 0.9 0.2 ± 0.1	0.8 ± 0.2 0.4 ± 0.2

Values are mean ± standard deviation or n (%). TAPSE: tricuspid annular plane excursion; PASP: pulmonary artery systolic pressure; RV: right ventricle; LV: left ventricle.

## Data Availability

The data presented in this study are available on request from the corresponding author. The data are not publicly available due to privacy.
